# Fecal indicators and antibiotic resistance genes exhibit diurnal trends in the Chattahoochee River: Implications for water quality monitoring

**DOI:** 10.3389/fmicb.2022.1029176

**Published:** 2022-11-10

**Authors:** Karena. H. Nguyen, Shanon Smith, Alexis Roundtree, Dorian J. Feistel, Amy E. Kirby, Karen Levy, Mia Catharine Mattioli

**Affiliations:** ^1^Department of Biology, Emory University, Atlanta, GA, United States; ^2^Rollins School of Public Health, Emory University, Atlanta, GA, United States; ^3^Waterborne Disease Prevention Branch, Division of Foodborne, Waterborne, and Environmental Diseases, National Center for Emerging and Zoonotic Infectious Diseases, Centers for Disease Control and Prevention, Atlanta, GA, United States; ^4^Department of Environmental and Occupational Health Sciences, University of Washington, Seattle, WA, United States

**Keywords:** fecal indicator bacteria, microbial source tracking markers, antibiotic resistance genes, Chattahoochee River, diurnal variability, sample collection, water quality

## Abstract

Water bodies that serve as sources of drinking or recreational water are routinely monitored for fecal indicator bacteria (FIB) by state and local agencies. Exceedances of monitoring thresholds set by those agencies signal likely elevated human health risk from exposure, but FIB give little information about the potential source of contamination. To improve our understanding of how within-day variation could impact monitoring data interpretation, we conducted a study at two sites along the Chattahoochee River that varied in their recreational usage and adjacent land-use (natural versus urban), collecting samples every 30 min over one 24-h period. We assayed for three types of microbial indicators: FIB (total coliforms and *Escherichia coli*); human fecal-associated microbial source tracking (MST) markers (crAssphage and HF183/BacR287); and a suite of clinically relevant antibiotic resistance genes (ARGs; *blaCTX-M, blaCMY, MCR, KPC, VIM, NDM*) and a gene associated with antibiotic resistance (*intl1*). Mean levels of FIB and clinically relevant ARGs (*blaCMY* and *KPC*) were similar across sites, while MST markers and *intI1* occurred at higher mean levels at the natural site. The human-associated MST markers positively correlated with antibiotic resistant-associated genes at both sites, but no consistent associations were detected between culturable FIB and any molecular markers. For all microbial indicators, generalized additive mixed models were used to examine diurnal variability and whether this variability was associated with environmental factors (water temperature, turbidity, pH, and sunlight). We found that FIB peaked during morning and early afternoon hours and were not associated with environmental factors. With the exception of HF183/BacR287 at the urban site, molecular MST markers and *intI1* exhibited diurnal variability, and water temperature, pH, and turbidity were significantly associated with this variability. For *blaCMY* and *KPC*, diurnal variability was present but was not correlated with environmental factors. These results suggest that differences in land use (natural or urban) both adjacent and upstream may impact overall levels of microbial contamination. Monitoring agencies should consider matching sample collection times with peak levels of target microbial indicators, which would be in the morning or early afternoon for the fecal associated indicators. Measuring multiple microbial indicators can lead to clearer interpretations of human health risk associated with exposure to contaminated water.

## Introduction

Fecal indicator bacteria (FIB), which include fecal coliforms, enterococci, and *Escherichia coli*, among other organisms, are commonly used by state and local environmental agencies as markers of elevated fecal contamination levels in water. FIB have been a critical tool for monitoring water bodies and minimizing health risk from water exposure through recreation, drinking, and agricultural irrigation (Sadowsky and Whitman, 2011). The United States Environmental Protection Agency (USEPA) and state agencies determine the thresholds for FIB in regulated water bodies, and exceedances are assumed to indicate a change in water quality and likely elevated human health risk, leading to boil-water advisories, beach closures, or other regulatory actions (USEPA, 2012).

State agencies predominantly culture FIB from 100 ml water samples and use these data to provide public health advisories to stakeholders (e.g., kayakers, beachgoers) in addition to regulatory action. However, research has demonstrated that in many untreated surface water bodies, the levels of FIB poorly predict the risk of gastrointestinal illness (Colford et al., 2007; Nelson et al., 2012; Arnold et al., 2013; Gruber et al., 2014) and do not correlate with the presence of water-transmitted pathogens of concern, such as enteric viruses (Harwood et al., 2005; Sinigalliano et al., 2010; Wu et al., 2011; Korajkic et al., 2018). The environmental persistence of FIB relative to pathogens of interest are also affected by UV radiation (Sinton et al., 2002; Walters et al., 2009; Nguyen et al., 2015), precipitation (Bower et al., 2005; Shehane et al., 2005; Tornevi et al., 2014; Mattioli et al., 2015; Harris et al., 2018), tidal cycles (Mill et al., 2006; Rosenfeld et al., 2006), and other environmental factors (Byappanahalli et al., 2012; Rochelle-Newall et al., 2015; Lee et al., 2018).

FIB also display high spatial and temporal variation in marine and fresh waters (Boehm et al., 2002; Boehm, 2007; Levy et al., 2009). For example, a 24-h study found that FIB concentrations in urban sub-catchment streams were significantly higher during daytime than nighttime hours (Ekklesia et al., 2015), suggesting that there may be times of the day where exposure to contaminated water could lead to a higher likelihood of pathogen exposure and health risks. The effectiveness of management decisions therefore depends on whether sample collection coincides with peak levels of FIB, and how strongly FIB positively correlate with pathogens of concern (Enns et al., 2012).

Given that human fecal contamination carries the greatest risk to human health (Soller et al., 2010), microbial source tracking (MST) has emerged as a complementary approach to water quality monitoring. MST markers can discriminate between potential sources of fecal contamination by targeting genes of host-associated fecal bacteria (Harwood et al., 2014). Human-associated MST markers, such as crAssphage (Stachler et al., 2017) and HF183/BacR287 (Green et al., 2014; USEPA, 2019), have demonstrated high sensitivity and specificity for human sewage in Canada, Costa Rica, Germany, and the United States (Ahmed et al., 2009; Stachler et al., 2018; Symonds et al., 2018). MST markers can help direct monitoring strategies by discriminating between probable sources of contamination, in turn improving health risk estimates, and by determining appropriate mitigation efforts when FIB levels are high (McQuaig et al., 2012; Nguyen et al., 2018).

Besides enteric viruses, bacteria, and other pathogens of concern, urban runoff and treated wastewater effluent can introduce antibiotic resistant organisms (AROs) and antibiotic resistance genes (ARGs) into receiving water bodies (Sanderson et al., 2016; Almakki et al., 2019; Karkman et al., 2019). Sewage spills (Young et al., 2016), heavy rainfall (Young et al., 2013; Ahmed et al., 2018; Stange and Tiehm, 2020), and other weather or anthropogenic events can further contaminate surface waters by releasing high concentrations of AROs and ARGs. Recent work suggests that ARGs of clinical significance could be used as microbial indicators of wastewater contamination (Zhang et al., 2020). However, there is a lack of data demonstrating the extent to which ARGs exhibit diurnal variability in water bodies that consistently receive urban runoff and/or treated wastewater effluent or how environmental factors are associated with the concentration of these genes in surface waters.

Understanding how microbial indicators vary over space and time could improve our ability to assess human health risk, as the co-occurrence of elevated FIB, human-associated MST markers, and ARGs could signify a much higher exposure risk than the detection of any of these microbial indicators alone. Using FIB in combination with MST markers can improve water quality monitoring and assessment in marine (Symonds et al., 2017) and fresh waters (Nguyen et al., 2018), but the relationship among FIB, MST markers, and antibiotic resistance in the aquatic environment remains understudied. It is therefore important to examine the concentration and variability of these microbial indicators concurrently, with different potential fecal inputs (e.g., natural versus urban), and within shorter time periods. Specifically, understanding within-day variability can provide critical information about better or worse times of the day for humans to recreate in or draw from water sources, and can inform water monitoring sample strategies to protect human health.

The Chattahoochee River receives both untreated urban runoff and treated wastewater effluent and also serves as the principal source of drinking water for the greater Atlanta metropolitan area with a population of approximately five million people (Gregory and Frick, 2001). In 2000, the National Park Service, U.S. Geological Survey (USGS), local and state agencies, and nongovernmental organizations founded the BacteriALERT program to monitor *E. coli* levels at three major sites in the Chattahoochee River. Weekly monitoring from 2000 to 2008 showed that during stormflow conditions at the urban Atlanta location (Paces Ferry), levels of *E. coli* routinely exceeded USEPA recreational water quality criteria of 126 colony forming units (CFU) per 100 ml of water (Lawrence, 2012; USEPA, 2012). Subsequent work has shown that septic systems also contribute to fecal pollution in this portion of the watershed (Sowah et al., 2014).

To gain a holistic assessment of exposures to microorganisms associated with negative health outcomes and to inform monitoring approaches in the Chattahoochee River, we conducted a high-resolution temporal study at two sites along the river that varied in their recreational usage and surrounding land use (natural versus urban), collecting samples every 30 min over one 24-h period. We assayed samples for culturable FIB (total coliforms and *E. coli*), human fecal-associated MST molecular markers (crAssphage and HF183/BacR287), and a suite of clinically relevant antibiotic resistance genes (*blaCTX-M, blaCMY, MCR, KPC, VIM, NDM*) and the mobile element class 1 integron gene associated with antibiotic resistance (*intl1*) [hereafter referred together as antibiotic resistance-associated genes (ARGs)]. Our primary objectives were to: (a) quantify overall levels of contamination between sites with distinct public water use and surrounding land uses; (b) examine correlations between FIB, MST markers, and ARGs; (c) determine the diurnal variability of FIB, MST markers, and ARGs between sites; and (d) evaluate the relationship between environmental factors and diurnal variability between sites.

## Materials and methods

### Site description and sample collection

The study was conducted at a natural (Cochran Shoals) and urban (Paces Ferry) site along the Chattahoochee River (Supplementary Figure S1). Cochran Shoals is located northwest of Atlanta, Georgia, United States in the Chattahoochee River National Recreation Area, a protected area with high recreational use (e.g., kayaking, swimming, dog walking; N33.902734, W84.442128). Paces Ferry is located northwest of Atlanta, Georgia, United States, downstream of the Cochran Shoals site and downstream of a combined sewer overflow (CSO; N33.859063, W84.454837), as well as residential homes, restaurants, and schools. Cochran Shoals and Paces Ferry will be henceforth referred to as the natural and urban sites, respectively. Research activities were approved by the U.S. Department of Interior National Park Service under permit CHAT-2016-SCI-0003 (study number CHAT-00124).

Water samples were collected at the shoreline of each site in 30-min intervals for 24 h from 04:00 on April 9, 2016 to 03:30 on April 10, 2016 for a total of 48 samples per site. At each interval, ~1 L of water was collected in a Whirl-pak bag (Nasco, Fort Atkinson, WI) at ankle-deep depth and placed on ice. Water temperature and pH were recorded at each sampling event with a WaterproofPocket pH Test (Hanna Instruments, Smithfield, RI). Water turbidity was also measured on-site using a 2100Q Portable Turbidimeter (Hach Company, Loveland, CO). Because sunlight is known to impact the environmental persistence of FIB and MST markers, Global Horizontal Irradiance (GHI) measurements (W/m^2^) were extracted from the National Solar Radiation Database^1^ on December 14, 2020. GHI measurements were extracted for the same half-hour increments of the sampling period and will be henceforth referred to as UV.

### Enumeration of fecal indicator bacteria

Samples were processed within 5 h of collection using the Colilert most probable number (MPN) method with IDEXX QuantiTray/2000 assays (IDEXX Laboratories, Inc. Westbrook, ME) to enumerate total coliforms and *E. coli* per 100 ml (USEPA, 2014). The range of quantification for the assay was 1–2,419 MPN/100 ml. Field and lab blanks were conducted for each site to ensure lack of cross contamination of samples during collection, transport, storage, and processing. Field blanks consisted of 1 L sterile, deionized water in a Whirl-Pak bag carried into the field during and then back to the lab for processing alongside samples. Lab blanks consisted of 100 ml sterile, deionized water processed for Colilert, or 250 ml sterile, deionized water membrane filtered, alongside samples to ensure culture media sterility and lack of molecular contamination for FIB and MST enumeration, respectively.

### Molecular methods

#### DNA extraction and quantitative polymerase chain reaction

For molecular assays, 250 ml of each sample were filtered through 0.45 μm-pore size nitrocellulose filters (HA type filters, Millipore, Billerica, MA) and stored at −80°C until extraction. DNA was extracted from filters using the Qiagen DNeasy PowerWater extraction kit (Catalog No. 14900-100-NF) according to manufacturer instructions. Briefly, filters were subject to bead beating lysis and processed with a final nucleic acid elution volume of 100 μl. All extracts were stored at −80°C until qPCR assays were performed.

Two MST markers, crAssphage target 056 (Stachler et al., 2017; Willis et al., 2022) and HF183/BacR287 (USEPA, 2019), were assayed as indicators of human fecal contamination. Markers of and indicators associated with ARGs were also assayed, including an environmentally common proposed AR-indicator gene (*intI1*; Barraud et al., 2010), beta-lactamase resistance genes known to be present in the Chattahoochee River (*blaCTX-M, blaCMY*; Birkett et al., 2007; Agga et al., 2015), and carbapenemase resistance genes of emerging concern (*MCR, KPC, VIM, NDM*; Ellington et al., 2016; Yang et al., 2017). Primer and probe sequences and concentrations for each qPCR assay are listed in the SI (Supplementary Table S1).

Briefly, each sample was analyzed by qPCR in triplicate using 2 μl of extract in 50-μl reactions using TaqPath™ qPCR Master Mix (Life Technologies, Grand Island, N) for detection of the AR-associated genes due to the necessity for high purity, ARG-free polymerase. For the MST markers, 2 μl of extract in 25-μl reactions using Environmental MasterMix 2.0 (Life Technologies, Grand Island, NY; MST) on an Applied Biosystems (ABI) 7,500 thermocycler (ABI, Carlsbad, CA). The thermal cycling parameters for all qPCR assays were as follows: 10 min denaturation at 95°C and then 45 cycles of 95°C for 15 s and 60°C for 1 min. To test for amplification inhibition, the HF183/BacR287 qPCR was run with an internal amplification control (Green et al., 2014; USEPA, 2019), and inhibition presence was defined as a Cq difference of more than 2.3 for the internal control spiked into the same versus the no template control (NTC) (Borchardt et al., 2021). For each instrument run, the amplification threshold was set to 0.03 for consistent comparison across runs. To monitor for extraneous DNA contaminants, three no template controls were included with each instrument run.

#### Molecular marker enumeration

A linearized synthetic DNA plasmid was used as a reference standard (Integrated DNA Technologies, Coralville, IA) to enumerate the genetic markers consisting of six 10-fold serial dilutions (10^0^–10^5^ copies per reaction; Table 1). For all qPCR assays, the limit of detection (LOD) and limit of quantification (LOQ) were determined according to published methods (Shanks et al., 2016; Forootan et al., 2017; Klymus et al., 2020). Samples were then designated as not detected (ND) or quantifiable. Samples with fewer than three replicate qPCR detections were designated as ND. Samples where all replicates were detectable and the mean number of gene copies per reaction exceeded the LOQ were designated as quantifiable. We estimated the concentration in quantifiable samples using the following equation:

**Table 1:** Summary of standard curve material and performance for the microbial source tracking (MST) markers and antibiotic resistance-associated genes (ARGs) quantified in the study.

Target^a^	Microbial indicator type	Efficiency	Slope	Intercept	*R* ^2^	LOD^b^ (gene copies/100 ml, ±SE)	LOQ^a^ (gene copies/100 ml)	Samples below LOD (%)
crAssphage	MST	96.84	−3.40	42.04	0.99	<10	10	6/93 (7%)
HF183/BacR287	MST	95.30	−3.44	37.62	0.99	14.81 (±2.79)	100	7/93 (8%)
*blaCMY*	ARG	94.54	−3.46	39.66	0.99	<10	10	27/93 (29%)
*intI1*	ARG	102.65	−3.26	42.79	0.99	69.47 (±64.64)	100	4/93 (4%)
*KPC*	ARG	98.44	−3.36	44.24	0.99	99.29 (±128.09)	100	50/93 (54%)

a Linearized synthetic DNA standard material (Integrated DNA Technologies, Coralville, IA): Willis et al. (2022) for crAssphage; Green et al. (2014) for HF183/BacR287; see SI for ARG standard information.

b LOD, limit of detection; LOQ, limit of quantification; SE, standard error.


(1) $$\frac{\frac{\mathrm{Extraction volume}}{\mathrm{Template volume}}*\mathrm{gene copies}/\mathrm{reaction}}{\frac{\mathrm{volume processed}}{100}}$$

Extraction volume refers to the volume of water that was membrane filtered (250 ml), template volume refers to the amount of template used in each well (2 μl), gene copies per reaction refers to the raw quantity from the qPCR assay, and volume processed refers to the volume of template after DNA extraction (100 μl). We standardized concentrations as gene copies per 100 ml of water.

### Statistical analyses

Statistical analyses were conducted using R 4.1.0 (R Core Team, 2021). The log_10_ means of FIB levels over the entire study period were compared between sites using two-sided *t*-tests. For crAssphage, HF183/BacR287, *blaCMY*, *intI1*, and *KPC* the mean concentration per triplicate qPCR was calculated, and then the log_10_ mean for each target was calculated over the entire study period and compared between sites using a two-sided *t*-test. In cases where the variance was unequal (i.e., crAssphage and *intI1*), a Welch’s *t*-test was conducted instead. Non-detected samples were excluded from two-sided and Welch’s *t*-tests to avoid artificially minimizing the standard error. Next, associations between FIB and MST markers, FIB and ARGs, and MST markers and ARGs at each site independent of environmental factors (i.e., temperature, pH, turbidity, and UV) were evaluated using Kendall’s non-parametric measure of correlation. Here, non-detected samples were set to half the LOD for each qPCR assay so the analysis could account for tied ranks.

The diurnal variability of total coliforms, *E. coli*, crAssphage, HF183/BacR287, *blaCMY*, *intI1*, and *KPC* was evaluated using Generalized Additive Mixed Models (GAMMs). Specifically, GAMMs were used to determine if the measured microbial indicators exhibited significant diurnal variability and how strongly environmental factors (i.e., water temperature, turbidity, pH, and UV) were associated with this variability. GAMMs facilitate inferences about nonlinear dynamics over time through the incorporation of smooth functions as predictors (Simpson, 2018). In the GAMMs, non-detected samples were set to half the LOD for each qPCR assay so that the temporal trends were not weighted toward non-detected (i.e., zero) samples.

Two separate models were fit for each environmental indicator (i.e., total coliforms, *E. coli*, crAssphage, HF183, *blaCMY*, *intI1*, and *KPC*) using the *gamm* function in the *mgcv* package (Wood, 2017; R Core Team, 2021). The first model tested for diurnal variability within a site, and if present, whether diurnal variability differed between sites. Model variables included: (1) a smooth temporal trend to estimate diurnal variation in the time series, (2) a “difference” smooth trend to identify unique temporal trends between the two sites, and (3) a random effect intercept for continuous autocorrelated errors at each site (*corAR1* function, Wood, 2017; R Core Team, 2021) to account for repeated sampling. The second model tested whether the environmental factors impacted diurnal variability within a site and if trends in diurnal variability were different between sites. Model variables included: (1) water temperature, (2) pH, (3) turbidity, (4) UV, (5) a smooth temporal trend to estimate diurnal variation in the time series, (6) a “difference” smooth trend to identify unique temporal trends between the two sites, and (7) a random effect intercept for continuous autocorrelated errors at each site (*corAR1* function, Wood, 2017; R Core Team, 2021) to account for repeated sampling. For each microbial indicator, we graphed the model with the highest adjusted *R^2^* value. In cases where the adjusted *R^2^* values were similar, we excluded the effect of environmental factors because no additional variation in diurnal variability was explained. For each microbial indicator, best fit model predictions and 95% confidence intervals are presented (*plot_smooth* function in the *itsadug* package, R Core Team, 2021; van Rij et al., 2022).

## Results

### Levels of microbial targets

FIB were consistently detected throughout the sampling period. Of the MST markers and ARGs tested in this study, only crAssphage, HF183/BacR287, blaCMY, intI1, and KPC were detected. Targets were never detected in field or laboratory blanks. The LOD, LOQ, and associated standard curve material and performance for assays are included in Table 1 (assays with no detected samples are excluded).

We found no significant difference in total coliforms (*t* = 0.29, *p* = 0.77), *E. coli* (*t* = 0.80, *p* = 0.43), *blaCMY* (*t* = 0.90, *p* = 0.37), and *KPC* (*t* = 1.05, *p* = 0.30) mean concentrations between the natural and urban sites (Table 2). However, mean concentrations of crAssphage (*t* = 4.35, *p* < 0.001), HF183/BacR287 (*t* = 2.84, *p* = 0.0057), and *intI1* (*t* = 2.19, *p =* 0.034) were significantly higher at the natural site compared to the downstream urban site (Table 2).

**Table 2:** Comparison of fecal indicator bacteria (FIB; CFU per 100 ml), microbial source tracking (MST) markers (copies per 100 ml), and antibiotic resistance-associated genes (ARGs; copies per 100 ml) between the natural (Cochran Shoals) and urban (Paces Ferry) sites.

Target	Log_10_ (Mean ± SE) (natural)	Log_10_ (Mean ± SE) (urban)	*t*	*df*	*p*
Total coliforms	2.39 ± 0.08	2.35 ± 0.10	0.29	92	0.77
*E. coli*	1.36 ± 0.05	1.30 ± 0.06	0.80	92	0.43
crAssphage	3.00 ± 0.03	2.69 ± 0.07	4.35	51.10	**<0.001**
HF183/BacR287	3.26 ± 0.06	3.03 ± 0.06	2.84	84	**0.0057**
*blaCMY*	3.36 ± 0.05	3.28 ± 0.06	0.90	64	0.37
*intI1*	6.48 ± 0.02	6.26 ± 0.10	2.19	43.50	**0.034**
*KPC*	3.07 ± 0.03	3.03 ± 0.02	1.05	41	0.30

A Welch’s *t-*test was applied for groups with unequal variance. *P*s are bolded for significance (*p* < 0.05).

### Correlation between microbial indicators

Associations between FIB and MST markers, FIB and ARGs, and MST markers and ARGs are presented in Table 3. At the natural site, we observed moderately positive correlations between total coliforms and HF183/BacR287, *blaCMY*, and *KPC* (*τ* range: 0.21–0.31). *Escherichia coli* had moderately positive correlations with all MST markers and ARGs (*τ* range: 0.31–0.57). CrAssphage and HF183/BacR287 most strongly positively correlated with *intI1* (*τ* = 0.53) and moderately correlated with *blaCMY* and *KPC* (*τ* range: 0.24–0.47; Table 3).

**Table 3:** Summary of correlations between fecal indicator bacteria (FIB; total coliforms and *E. coli*), microbial source tracking (MST) markers (crAssphage and HF183/BacR287), and antibiotic resistance-associated genes (ARGs; *blaCMY*, *intI1*, and *KPC*) between the natural (Cochran Shoals) and urban (Paces Ferry) sites.

		**crAssphage**	**HF183/BacR287**	***blaCMY***	***intI1***	***KPC***
Natural	Total coliforms	0.16	**0.31**	**0.33**	0.15	**0.21**
	*E. coli*	**0.31**	**0.57**	**0.37**	**0.39**	**0.35**
	crAssphage			**0.34**	**0.53**	**0.24**
	HF183/BacR287			**0.41**	**0.53**	**0.33**
Urban	Total coliforms	−0.0067	0.13	−0.01	−0.20	−0.05
	*E. coli*	**−0.24**	0.06	**−0.24**	**−0.24**	**−0.25**
	crAssphage			**0.46**	**0.59**	**0.47**
	HF183/BacR287			**0.41**	**0.55**	**0.41**

Tau values with significant *P*s (*p* < 0.05) are bolded.

At the urban site, total coliforms did not correlate with MST markers or ARGs. *Escherichia coli* negatively correlated with crAssphage, *blaCMY*, *intI1*, and *KPC* (*τ* range: −0.24 to −0.25). Similar to trends at the natural site, crAssphage and HF183/BacR287 most strongly correlated with *intI1* (*τ* range: 0.55–0.59) and also moderately correlated with *blaCMY* and *KPC* (*τ* range: 0.41–47).

### Diurnal variability and association with environmental factors

**Figure 1:**
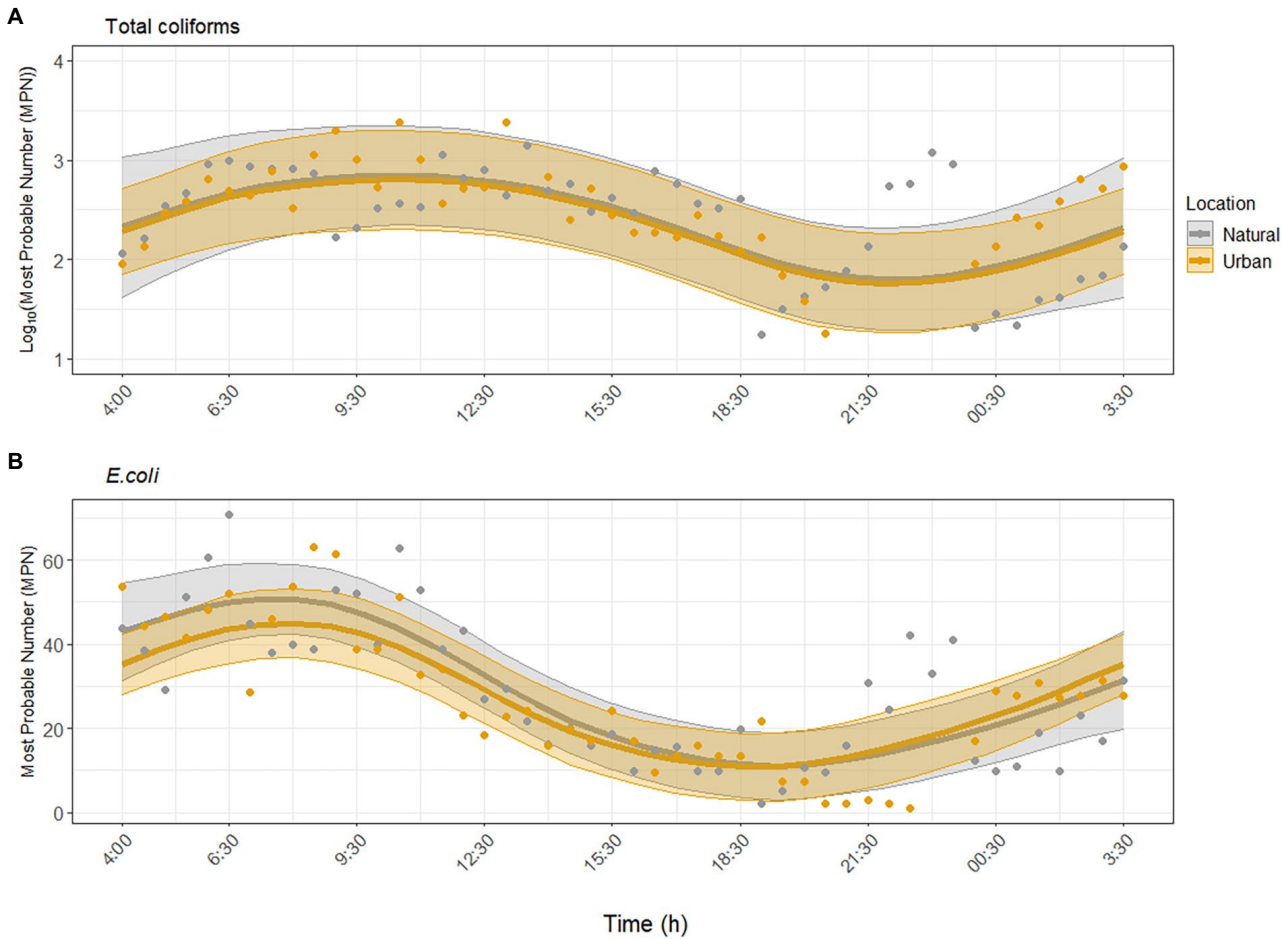
Dynamics of fecal indicator bacteria **(A)** total coliforms and **(B)** *Escherichia coli* over the sampling period at the urban (Paces Ferry) and natural (Cochran Shoals) sites independent of environmental factors. Solid lines represent model predictions with 95% confidence intervals. Points represent detected samples.

Environmental parameters measured at both sites during the study period are provided in the SI (Supplementary Figures S2–S4). Total coliforms and *E. coli* were consistently detected and showed similar nonlinear, temporal trends at both sites (Figure 1). Specifically, FIB peaked between 08:00 and 12:30 and were lowest between 20:00 and 00:30. Concentrations of total coliforms ranged between 2.0–2.4 × 10^3^ MPN/100 ml across sites, and *E. coli* concentrations ranged between 1 and 70.8 MPN/100 ml. GAMM analysis showed that total coliforms and *E. coli* exhibited significant diurnal variability at the urban site (*p* = 0.004; *p* < 0.001), and there was no significant difference in the diurnal variability trend at the natural site (*p* = 0.98, adjusted *R^2^* = 0.43, Figure 1A; *p* = 0.40, adjusted *R^2^* = 0.67, Figure 1B). Including water temperature, pH, turbidity, and UV did not improve the model fit; thus these environmental factors were not significantly associated with the diurnal variability of total coliforms and *E. coli* (Supplementary Tables S1, S2).

**Figure 2:**
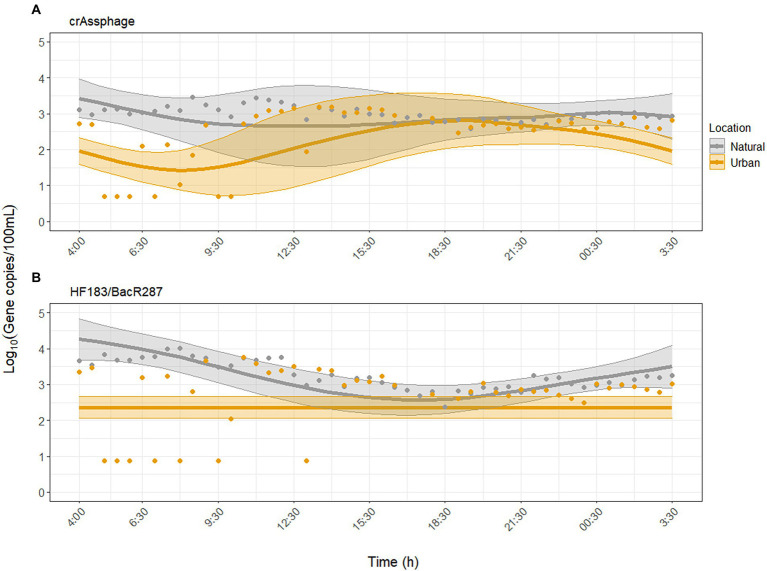
Dynamics of microbial source tracking markers **(A)** crAssphage and **(B)** HF183/BacR287 over the sampling period at the urban (Paces Ferry) and natural (Cochran Shoals) sites. Water temperature, pH, and turbidity were significant predictors of diurnal variability for both MST markers. Solid lines represent model predictions with 95% confidence intervals. Points represent all collected samples, with undetected samples set to half the limit of detection (LOD) for each assay.

The human fecal-associated MST markers were inconsistently detected in the morning (until 10:00) at the urban site (Figure 2). For crAssphage, GAMM analysis independent of environmental factors showed that crAssphage did not exhibit diurnal variability at the urban site (*p* = 0.10) but did at the natural site (*p* = 0.007, adjusted *R^2^* = 0.31). When environmental factors were included as predictors, the model fit for crAssphage improved (adjusted *R^2^* = 0.62). Diurnal variability of crAssphage was significant at both sites (*p* < 0.001), but displayed significantly different trends (*p* < 0.001). In the second model, concentrations of crAssphage peaked between 17:00–21:30 at the urban site and between 04:00 and 09:30 at the natural site (Figure 2A). For crAssphage, water temperature, pH, and turbidity were significantly associated with diurnal variability, while UV was not (Supplementary Table S4).

For HF183/BacR287, GAMM analysis independent of environmental factors showed that HF183/BacR287 did not exhibit diurnal variability at the urban site (*p* = 0.87) but did at the natural site (*p* < 0.001, adjusted *R^2^* = 0.22). When we adjusted for the effect of environmental factors, the model fit improved (adjusted *R^2^* = 0.45), but diurnal variability was still not detected at the urban site (*p* = 0.07). At the natural site, concentrations peaked between 02:00–06:30 and declined from 06:30–18:30 (Figure 2B). Water temperature, pH, and turbidity were significantly associated with concentrations of HF183/BacR287, while UV was not (Supplementary Table S6).

**Figure 3:**
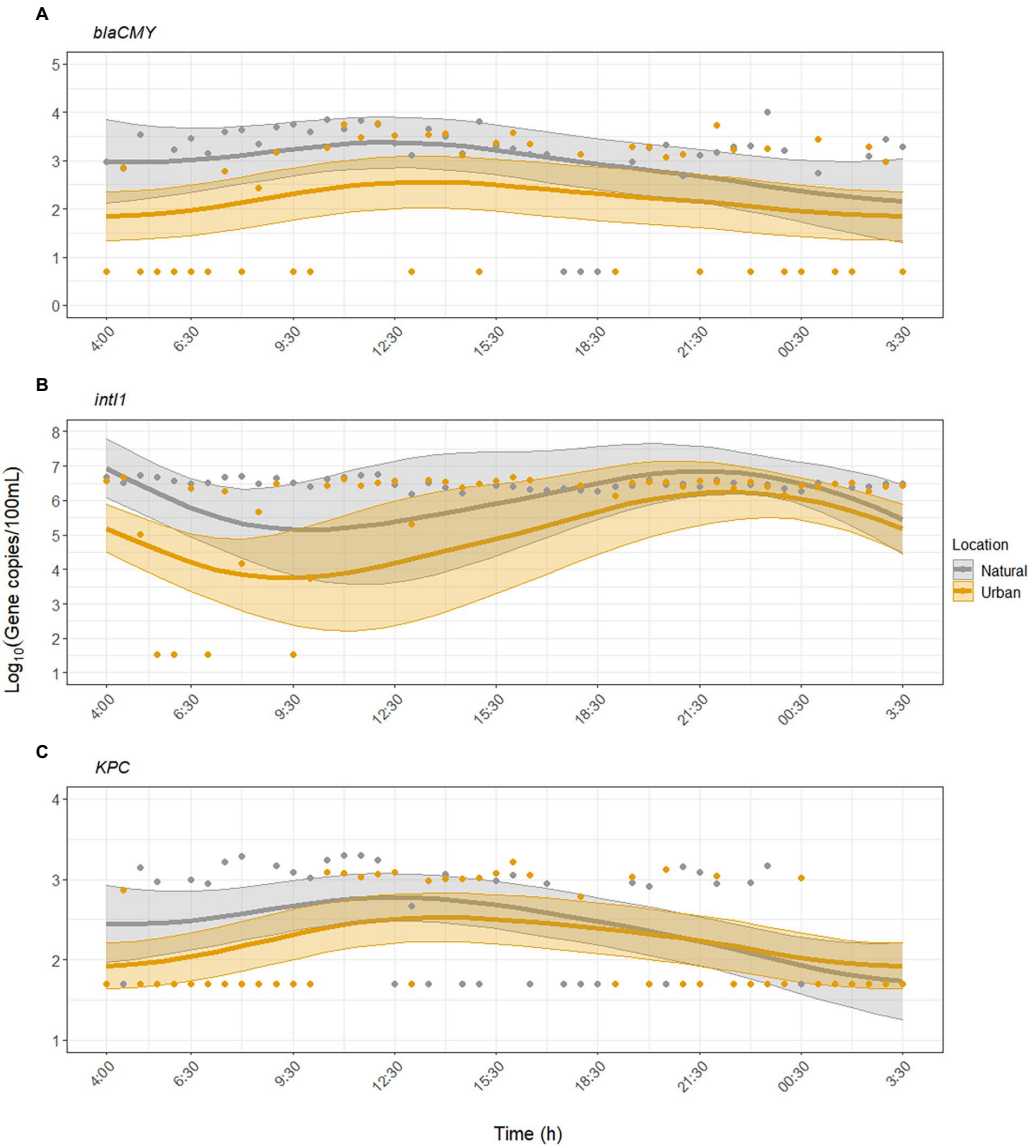
Dynamics of antibiotic resistance-associated genes **(A)** *blaCMY*, **(B)** *intI1*, and **(C)** *KPC* over the sampling period at the urban (Paces Ferry) and natural (Cochran Shoals) sites. Solid lines represent model predictions with 95% confidence intervals. The models for *blaCMY* and *KPC* do not include the effect of environmental factors. The model for *intI1* includes the effect of water temperature, pH, and turbidity. Points represent all collected samples, with undetected samples set to half the limit of detection (LOD) for each assay.

GAMM analysis showed that *KPC* and *blaCMY* exhibited significant diurnal variability but were inconsistently detected compared to FIB and MST markers (Figures 2, 3). For *blaCMY*, the urban site had undetected samples throughout the day (18/45 samples were below the LOD). Independent of environmental factors, GAMM analysis showed that *blaCMY* exhibited significant diurnal variability at the urban site (*p* = 0.043), as well as a significantly different diurnal trend at the natural site (*p* = 0.018, adjusted *R^2^* = 0.16). Concentrations of *blaCMY* peaked between 8:00 and 12:30 at both sites (Figure 3A). When adjusting for environmental factors, the model fit slightly improved (adjusted *R^2^* = 0.20). However, this model was weighted toward undetected samples and resulted in a loss of detection of diurnal variability (Supplementary Table S6); therefore, the model excluding the effect of environmental factors is shown in Figure 3A.

For *intI1*, GAMM analysis showed significant diurnal variability at the urban site (*p* = 0.003) and a different, significant diurnal trend at the natural site (*p* = 0.005, adjusted *R^2^* = 0.24). The model fit improved when we included the effect of environmental factors (adjusted *R^2^* = 0.38). At both sites, concentrations of *intI1* peaked between 6:30 and 12:30, matching the diurnal trends observed with crAssphage (Figure 3B). Water temperature, pH, and turbidity were significant predictors of diurnal variability while UV was not (Supplementary Table S7).

*KPC* was inconsistently detected at both sites (50/93 samples were below the LOD). GAMM analysis showed that *KPC* exhibited significant diurnal variability at the urban site (*p* = 0.003), with a similar significant diurnal trend at the natural site (*p* = 0.10, adjusted *R^2^* = 0.19). The temporal trend closely matched that of *blaCMY*, with peak concentrations of *KPC* occurring between mid-morning to early afternoon (maximum value occurred at 11:00, Figure 3C). The inclusion of environmental factors did not improve the model fit (adjusted *R^2^* = 0.19), indicating that environmental factors were not significantly associated with diurnal variability for *KPC* (Supplementary Table S8).

## Discussion

In this study, we evaluated the daily variation in levels of FIB, human-associated MST markers, and ARGs at two sites with different adjacent land use along the Chattahoochee River. We also examined correlations between FIB, MST markers, and ARGs to determine whether the concurrent use of these microbial indicators could capture high-risk human-specific organisms, and what information could be inferred from measures of individual microbial indicators.

### Overall levels of microbial indicators

We quantified maximum and mean levels of contamination at the natural and urban sites to assess whether FIB, MST markers, and ARGs occurred at concentrations that may be of human health concern. Throughout the sampling period, total coliforms and *E. coli* did not exceed 2.42 × 10^3^ MPN/100 ml and 70.80 MPN/100 ml, respectively. While these values are not directly comparable to monitoring thresholds that are typically based on 30-day geometric means, no single sample exceeded the 30-day geometric mean threshold of 126 CFU/100 ml of *E. coli* (The Department of Natural Resources Environmental Protection Division, 2021). Between sites, mean FIB levels were similar (Table 2), suggesting that varied usage and differences in likely contamination sources may not greatly impact FIB levels. This result is unsurprising, as FIB (e.g., fecal coliforms, enterococci, and *E. coli*) are shed in the feces of a variety endo- and ectotherms and are not animal specific (Field and Samadpour, 2007; Harwood et al., 2014).

Concentrations of crAssphage and HF183/BacR287 ranged from 10.9–2.94 × 10^3^ gene copies/100 ml and 1.10×10^2^–1.00×10^4^ gene copies/100 ml, respectively. These concentrations were one to two orders of magnitude lower than a previous study, which found between 10^4^–10^6^ gene copies/100 ml and 10^2^–10^5^ gene copies/100 ml of crAssphage and HF183/BacR287, respectively, in an urban stream directly downstream of a CSO (Stachler et al., 2018). No single sample in our study exceeded the recommended threshold of 4.6 × 10^3^ gene copies/100 ml of crAssphage as estimated by quantitative microbial risk assessment (QMRA; Crank et al., 2019), an approach that estimates concentrations of MST markers that correlate to 30 illnesses per 1,000 bathers. In contrast, 16 of 93 samples exceeded the recommended threshold of 4.2 × 10^3^ gene copies/100 ml of HF183/BacR287 as estimated by QMRA (Boehm et al., 2015). It should be noted, however, that there are currently no proposed thresholds for secondary natural exposures (e.g., wading, fishing, and boating) and risk of illness due to these types of exposures are understudied (Adhikary et al., 2022).

Maximum concentrations of *intI1*, *blaCMY*, and *KPC* were 5.69 × 10^6^, 1.00 × 10^4^, and 1.98 × 10^3^ gene copies/100 ml, respectively, and occurred at different times during the sampling period (Figure 3). *IntI1* occurred at higher mean concentrations at the natural site while *blaCMY* and *KPC* showed no significant differences in mean concentrations between sites (Table 2). Because there are currently no recommended thresholds for routine monitoring of ARGs, it is difficult to interpret these concentrations in terms of health risk due to exposure. However, the presence of these genes, which confer resistance to β-lactam antibiotics (Birkett et al., 2007; Agga et al., 2015), often signifies a greater risk to human health. As such, ARGs could also be used as microbial indicators in areas where human usage is high to monitor for sewage and antibiotic resistance contamination (Narciso-da-Rocha et al., 2014; Ishii, 2020; Zhang et al., 2020).

The consistent detection of FIB and human-associated MST markers throughout the day supports that extensive human fecal contamination is present in these waterways. However, we found significantly higher mean concentrations of crAssphage, HF183/BacR287, and *intI1* at the natural site, suggesting that there may be a heightened risk of illness at this site where humans are consistently recreating and contacting the water. The presence of ARGs at both sites also signals elevated human health risk and necessitates further evaluation.

### Correlation between microbial indicators

We found no consistent associations between the presence or concentration of total coliforms and the human-associated MST markers and ARGs. In contrast, *E. coli* levels were positively correlated with HF183/BacR287 and crAssphage at the natural site. CrAssphage and HF183/BacR287 levels were positively correlated with all intl1 and ARGs at the natural and urban sites. Our results support previous work that found positive correlations between crAssphage, HF183/BacR287, and culturable *E. coli* in an urban freshwater stream (Stachler et al., 2018). Our data are also in accordance with McKee et al. (2020), who conducted a 20-month survey of FIB and MST markers in the Chattahoochee River National Recreation Area and found that elevated *E. coli* levels positively correlated with dog and human-associated MST markers. In a separate study conducted by Stachler et al. (2019), crAssphage also positively correlated with ARGs in an urban stream, suggesting that crAssphage may indicate the presence of ARGs in polluted water bodies.

There were no significant associations between *E. coli* and any MST markers, AR indicators (intl1), or ARGs at the urban site. The lack of consistent associations between *E. coli* and MST markers in our study (Table 3) and others (Bradshaw et al., 2016) supports that differences in land use may not greatly impact overall levels of FIB but may lead to differences in fecal contamination and the subsequent detection of human- and wildlife-associated MST markers (Staley et al., 2013). The positive correlations we identified between the human-associated MST markers and ARGs also indicate that human fecal matter is likely present at both sites along the Chattahoochee River, and individuals may be at risk of exposure to AROs. In areas where human-associated MST markers are consistently detected, state agencies could consider monitoring ARGs and/or MST markers in addition to FIB for a more robust evaluation of potential health risks.

### Diurnal variability and association with environmental factors

At both sites, concentrations of total coliforms and *E. coli* peaked between 06:30 and 12:30 and decreased up to one order of magnitude throughout the day (Figure 1). There was no significant difference in diurnal variability between sites and environmental factors did not significantly impact diurnal variability. This result contrasts that of Aulenbach and McKee (2020), who found that turbidity was a positive predictor of *E. coli* in the Chattahoochee River. Previous work has shown that solar irradiation and indigenous microbiota may have the strongest effect on decreasing culturable FIB over time in marine waters (Mattioli et al., 2015; Wanjugi et al., 2016), but our results suggested negligible effects of environmental factors on FIB levels within a day in a flowing, freshwater river.

The human-associated MST markers exhibited significant differences in diurnal variation between sites. CrAssphage was relatively stable at the natural site and peaked between 17:00 and 21:30 at the urban site. HF183/BacR287 peaked in the morning at the natural site and showed no diurnal variability at the urban site (Figure 2). The diurnal variability of crAssphage at both sites was associated with water temperature, pH, and turbidity, but not UV (Supplementary Table S4). The diurnal variability of HF183/BacR287 at the natural site was associated with these same environmental factors, but not at the urban site, likely due to numerous non-detected samples. The urban site, which resides downstream of a CSO and is adjacent to runoff sites but had lower concentrations and fewer detections of crAssphage and HF183/BacR287 throughout the day compared to the natural site. A study conducted by Damashek et al. (2022) at 115 sites across the Upper Oconee watershed in Georgia found that highly contaminated waters were more strongly correlated with non-point sources (e.g., sewer density, septic system age) than CSOs. This study, along with our results, support that proximity to natural areas may place individuals at higher risk of exposure to human fecal contamination than previously assumed.

ARGs exhibited significant diurnal variation but varied in their detection rates as expected. Integrons are common genes in Gram-negative bacteria frequently detected in environmental waters (Barraud et al., 2010) and wastewater impacted waters (Agramont et al., 2020), and *intI1* occurred at concentrations two to three orders of magnitude greater than *blaCMY* and *KPC*. Diurnal variability of *intI1* peaked later in the day, possibly due to non-detected samples in the morning (Figure 3B), and was significantly associated with changes in water temperature, pH, and turbidity (Supplementary Table S7). In contrast, *blaCMY* and *KPC* were infrequently detected, and diurnal variability was not associated with environmental factors (Supplementary Tables S6, S8). Numerous non-detected samples throughout the day limited our ability to identify an effect of environmental factors on diurnal variability of ARGs. Therefore, additional research is needed to assess this hypothesis and whether other factors (e.g., rainfall) may have a greater impact.

To assess where individuals interacted with the water in our study area, we conducted structured observations at the natural site between 09:00 and 14:00 on four separate weekends in April 2016, including during the 24-h study period (see Supplementary material for detailed methods and observations). We found that most visitors interacted with the water through kayaks (32/36, 89%) or canoes (4/36, 11%), with limited contact with the water through wading (15/36, 42%) or standing (11/36, 31%). Other activities included swimming, splashing, and fishing. Few individuals touched their hands or face to the water. During the 24-h sampling period, dogs were also observed to swim in the river and owners had secondary contact with the water when touching their dogs (Supplementary Table S9). Studies have shown that canine fecal pollution can contribute to elevated FIB levels (Ervin et al., 2014; Riedel et al., 2015), and future studies could use canine-associated MST markers to determine if dogs are an additional source of fecal pollution at the natural site.

### Study limitations

The generalizability of our study is limited by the fact that samples were collected from the riverbank and not depth averaged throughout the water column according to standard methods (U.S. Geological Survey, 2006). However, this method is comparable to community scientist monitoring of surface waters, such as the Neighborhood Water Watch, a collaborative program between the Chattahoochee Riverkeeper organization and surrounding community members.^2^ The shoreline is also where humans typically have direct contact with the water, whereas within the stream, individuals are usually in a watercraft. The study was also limited by the fact that diurnal variation was only evaluated over 1 day. The conclusions of this study would be further strengthened by replication for multiple days during various seasons and rainfall conditions, but these preliminary data support that diurnal variation likely plays a role in the detectability of microbial indicators, especially FIB and MST markers. Another study limitation is that extraction blanks were not completed for the qPCR assays. While this is not ideal for determining if contamination occurred during extraction, field and laboratory blanks were processed and assayed for all targets, and no targets were detected in any of these blanks. Therefore, the field and laboratory blanks served as appropriate proxies for extractions blanks.

## Conclusion

Our data demonstrate the importance of sampling at high temporal resolutions for determining ideal monitoring times to understand potential health risk from water exposure. Both sites demonstrated evidence of human fecal contamination, but the maximum concentrations of FIB, MST markers, and ARGs occurred at different times of the day and did not align with each other. Therefore, monitoring of human MST markers and ARGs in human-impacted surface waters may give a more robust evaluation of human health risks from human shed pathogens and AROs than FIB alone (Ballesté et al., 2019; González-Fernández et al., 2021). Monitoring agencies should consider both surrounding land use and sample collection times to correspond with peak levels of target microbial indicators. Because it is difficult to interpret elevated levels of FIB in untreated surface waters as definitive evidence of recent fecal contamination events, FIB, human-associated MST markers, and/or ARGs can be used concurrently to maximize the understanding of health risk monitoring data.

## Data availability statement

The original contributions presented in the study are included in the article/Supplementary material, further inquiries can be directed to the corresponding author.

## Author contributions

KN led the data analysis and manuscript writing. SS led and conducted the field sampling. AR and DF conducted the molecular laboratory assays. AK supervised the ARG assay selection, execution, and interpretation. KL provided laboratory and supplies resources and served as a subject matter expert for data analysis and manuscript development. MM served as PI for the project and led study design and administration, field collection and processes, laboratory analyses, data analysis, and manuscript writing and submission. All authors contributed to the article and approved the submitted version.

## Conflict of interest

The authors declare that the research was conducted in the absence of any commercial or financial relationships that could be construed as a potential conflict of interest.

## Publisher’s note

All claims expressed in this article are solely those of the authors and do not necessarily represent those of their affiliated organizations, or those of the publisher, the editors and the reviewers. Any product that may be evaluated in this article, or claim that may be made by its manufacturer, is not guaranteed or endorsed by the publisher.

## Author disclaimer

The findings and conclusions of this paper are those of the authors and do not necessarily represent the official position of the U.S. Centers for Disease Control and Prevention (CDC).

## Footnotes

^1^ https://nsrdb.nrel.gov/

^2^ https://chattahoochee.org/our-work/water-quality-monitoring/
