# Supplementary material for: Fecal indicators and antibiotic resistance genes exhibit diurnal trends in the Chattahoochee River: Implications for water quality monitoring
Source: Front Microbiol. 2022 Nov 10;13:1029176. doi: 10.3389/fmicb.2022.1029176 (PMC9684717; doi:10.3389/fmicb.2022.1029176)
Supplement: Supplementary file 1 [file Data_Sheet_1.docx]

**Fecal indicators and antibiotic resistance genes exhibit diurnal trends in the Chattahoochee River: Implications for water quality monitoring**

**K. H. Nguyen^1^, S. Smith^2^, A. Roundtree^3^, Dorian J.** **Feistel^3^, A. E. Kirby^3^, K. Levy^2,4^, M.C. Mattioli^2,3^**^1^ Department of Biology, Emory University, Atlanta, GA, USA

^2^ Rollins School of Public Health, Emory University, Atlanta, GA, USA

^3^ Waterborne Disease Prevention Branch, Division of Foodborne, Waterborne, and Environmental Diseases, National Center for Emerging and Zoonotic Infectious Diseases, Centers for Disease Control and Prevention, USA

^4^ Department of Environmental and Occupational Health Sciences, University of Washington, Seattle, Washington, USA

**Corresponding author**:

M.C. Mattioli, kuk9@cdc.gov

**Supplemental Methods**

Sequence of purified, RNA-free gBlocks® Gene Fragment DNA standard reference material for ARG qPCR assays (Reference No. 198201051) (Integrated DNA Technologies, Coralville, IA):

ATGGAGCAAAACCCGCAGTCACAGCTGAAACTTCTTGTCACCCGTGGTAAGGAGCAAGGCTATCTGACCTATGCCGAGGTCAATGACCATCTGCCGGAAGATATCGTCGATTCAGATCAGATCGAAGACATCATCCAAATGATCAACGACCACTATTCTCAGAATGACTTGGTTGAGTACTCACCAGTCACAGAAAAGCATCTTACGGATGGCATGACAGTAAGAGAATTATGCATCGAGACGTTTAACGGCGTGTTGGGCGGCGATGCTATCGCCCGCGGCGAAATTAAGCTCAGCGATCCGGTCACGAAATACTGGCCAGAACTGACAGGCAAACAGTGGCAGGGTATCCGCCTGCTGCACTTAATCGGATTATGGTAATGAGGACATTTCGGGCAATGTAGACAGTTTCTGGCTCGACGGTGGTATTCGAATTTCGGCCACGGAGCAAATCAGCTTTTTAAGAAAGCTGTATCACAATAAGTTACACGTATCGGAGCGCAGCCAGCGTATTGTCAAACAAGCCATGCTGACCGAAGCCAATGGTGACTATATTATTCGGGCTAAAACTGGATACTCGACTAGAATCGAACCTAAGATTGGCTGGTGGGTCGGTTGGGTTGAACTTGATGATAATGTGTGGTTTTTTGCGATGAATATGGATATGATCGGCAGCGGCAGCAGTTTGTTGATTGGCTAAAGGGAAACACGACCGGCAACCACCGCATCCGCGCGGCGGTGCCGGCAGACTGGGCAGTCGGAGACAAAACCGGAACCTGCGGAGTGTATGGCACGGCAAATGACTATGCCGTCGTCTGGCCCACTGGGCGCGCACCTATTGTGTTGGCCGTCTACATCGCCAGCAAATGGAAACTGGCGACCAACGGTTTGGCGATCTGGTTTTCCGCCAGCTCGCACCGAATGTCTGGCAGCACACTTCCTATCTCGACATGCCGGGTTTCGGGGCAGTCGCTTCCAACGGTTTGATCGTCAGGGATGGCGGCCGCGTGCTGGTGGTCGATACCGCCTGGACCGATGACCAGACCGCCCAGATCCTCAACTGGATATCGCATCGCGGACAATCTCGGCTTTGTGCTGACGATCGCTGTCGTGCTCTTTGGCGCGATGCTACTGATCACCACGCTGTTATCATCGTATCGCTATGTGCTAAAGCCTGTGTTGATTTATCGTGATGGTATGCAACAAGTCGTAAATAAAACACATAAAGAAGATATTTATAGATCTTATGCAAACTTAATTGGCAAATCCGGTACTGCAGAACTCAAAATGAAACAAGGAGAAACTGGCAGACAAATTGGGTGGTTATCGTTGCCGATCGCGTGAAGTTCCGCCGCAAGGCTCGCTGGACCCAGATCCTTTACAGGAAGGCCAACGGATCGGCAGAGCGTGGTTCAGTCTGTTCGGGATAAGCTCTCCGCCGATATTATCATCAAGCAGACGGTGTCATCGCTGTGAGGTCGGTTGTGCGGTATTGGGAAACAGTGCCGCGTTAGTTGTTGGCGAGGTGGACCAAAATCGATGTGCAGCACCAGTAAAGTGATGGCCGCGGCCGCGGTGCTGAAGAAAAGTGAAAGCGAACCGAATCTGTTAAATCAGCGAGTTGAGATCAAAAAATCTGACCTTGTTAACTATAATCCGATTGCGGAAAAGCACGTCAATGGGACGATGTCACTGGCTGAGCTTAGCGCGGCCGCGCTACAGTACAGCGATAACGTGGCGATGAATAAGCTGATTGCTCACGTTGGCGGCCCGGCTAGCGTCACCGCGTTCGCCCGACAGCTGGGAGACGAAACGTTCCGTCTCGACCGTACCGAGCCGACGTTAAACACCGCCATTCCGGGCGATCCGCGTGATATCGGATCGGTCGAATGCGTGTGCTGCGCAAAAACCCAGAACCACGGCCAGGAATGCCCGGCGCGCGGATACTTCCGCTCAAGGGCGTCGGGAAGCGCAACGCCGCTGCGGCCCTCGGCCTGGTCCTTCAGCCACCATGCCCGTGCACGCGACAGCTGCTCGCGCAGGCTGGGTGCCAAGCTCTCGGGTAACATCAAGGCATCGTTGCTTTTGATTGATACAGCGTGGGGTGCGAAAAACACAGCGGCACTTCTCGCGGAGATTGAAAAGCAAATTGGACTTCCCGTAACGCGTGCAGTCTCCACGCACTTTCATGACGACCGCGTCGGCGGCGTTGATGTCCTTCGGGCGGCTGGGGTGGCAACGTACATCGATATGAACACCGACTACACGCTGGAAGAAGTGGGTAAACAGTTCGACGTTACCCGCGAACGTATCCGTCAGATCGAAGCGAAGGCGCTGCGCAAACTGCGTCACCCGAGCCGTTCTGAAGTGCTGCGTAGCTTCCTGGACGATTAA

**Supplemental Figures**


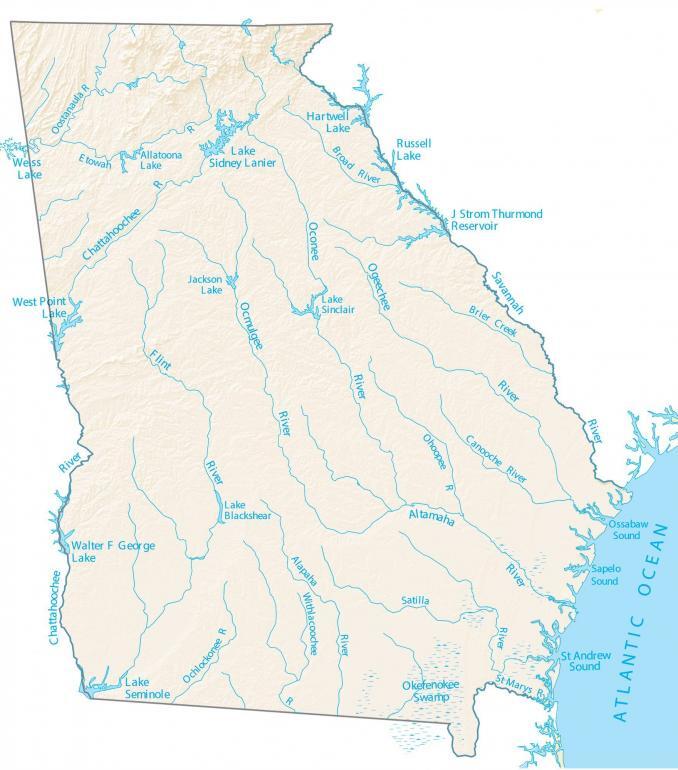

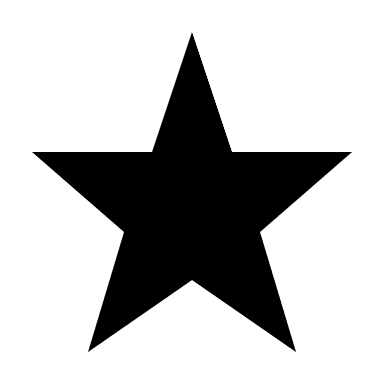

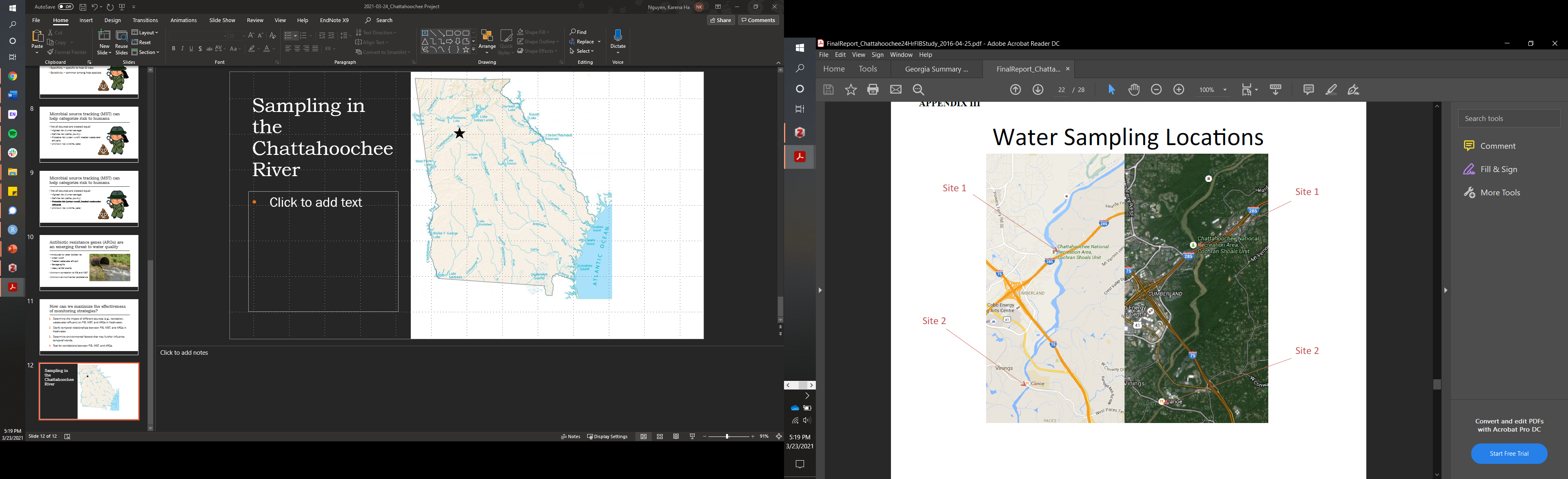


**Figure S1.** This map shows the sampling site locations, where site 1 is Cochran Shoals (natural) and site 2 is Paces Ferry (urban).^[[1]](#endnote-1)^

**
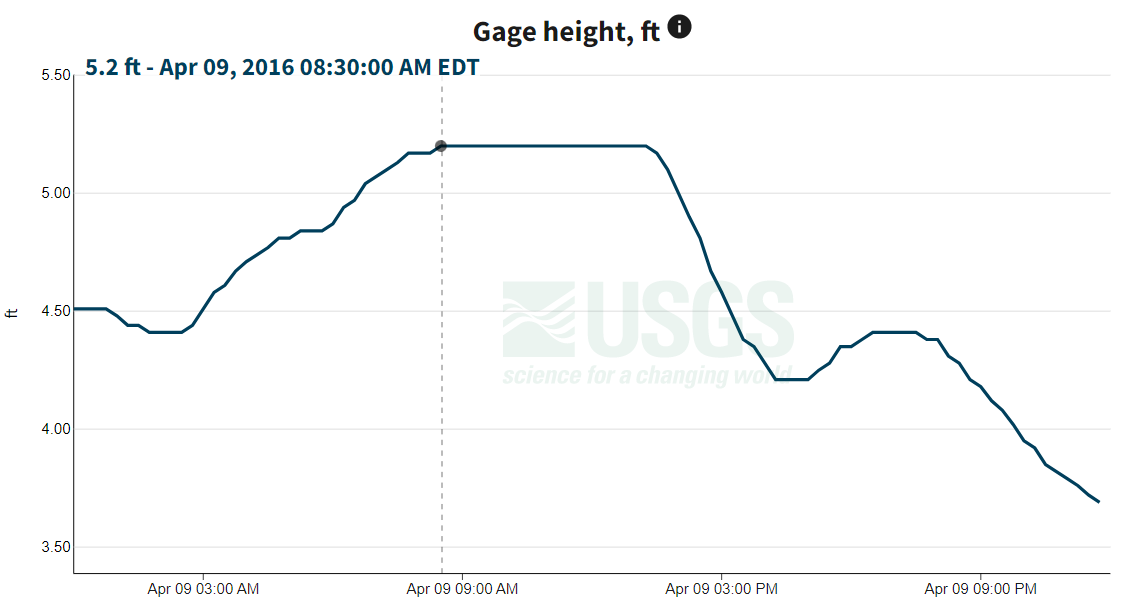
**

**Figure S2.** Chattahoochee River gage height during study period at Cochran Shoals (natural) site (USGS monitoring location 02335880). Peak flow occurred between 9:00 AM and 2:30 PM.**^[[2]](#endnote-2)^**

**
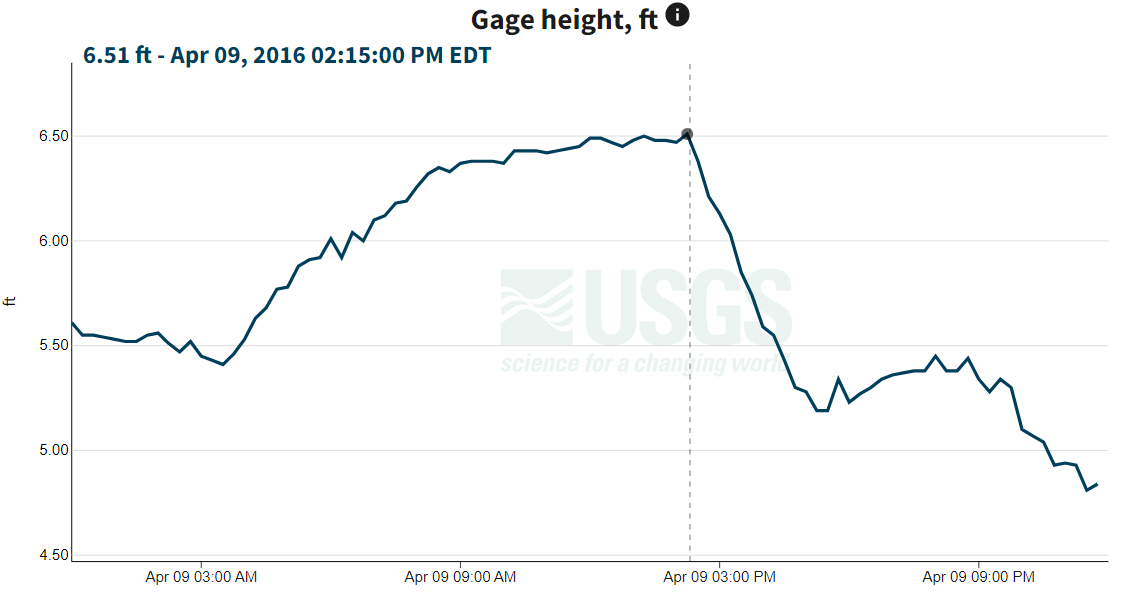
**

**Figure S3.** Chattahoochee River gage height during study period at Paces Ferry (urban) site (USGS monitoring location 02335990). Flow steadily increased until 2:30 PM.^ii^


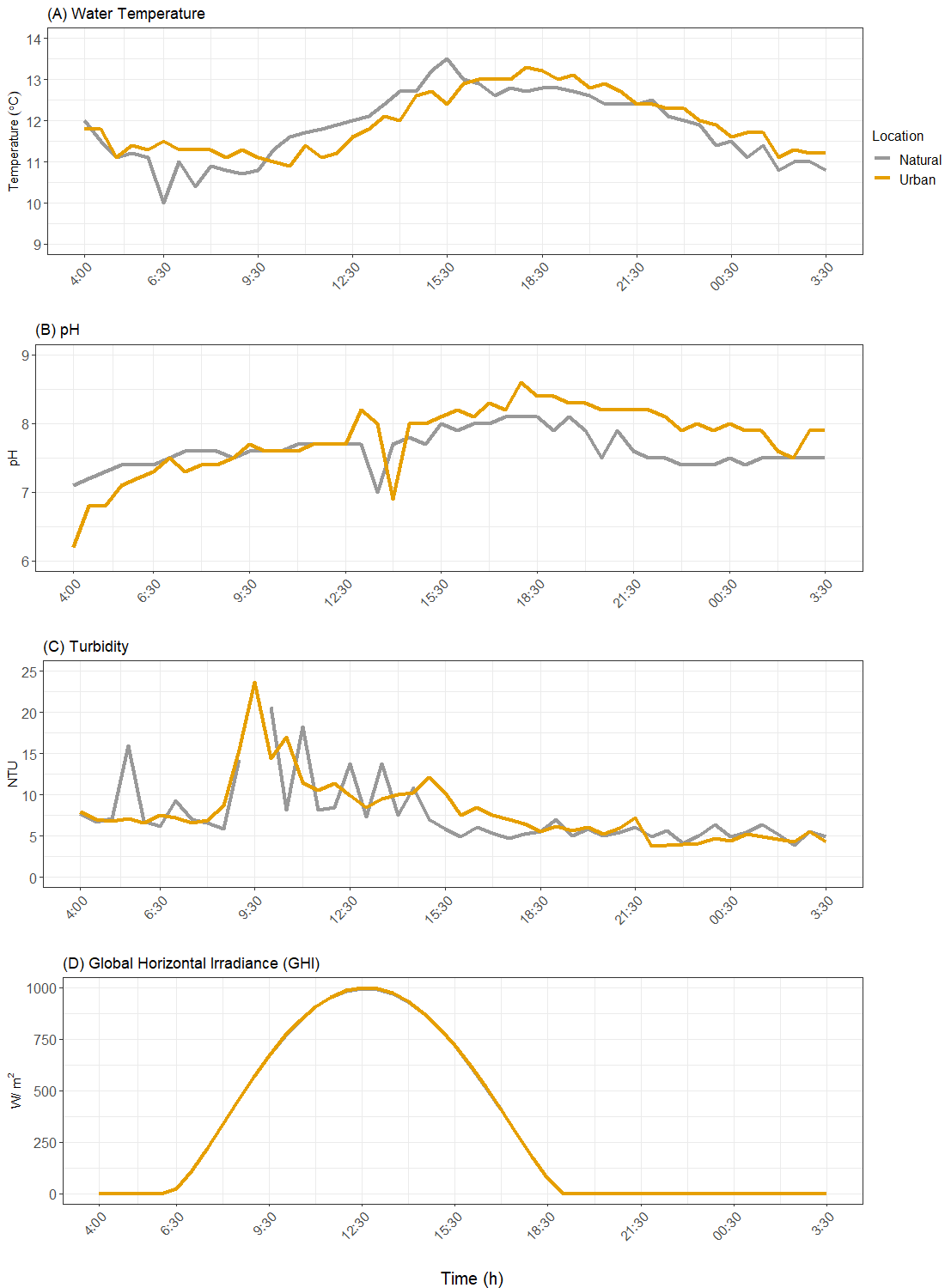


**Figure S4.** Time series of the environmental parameters measured in the field throughout the study period (April 9, 2016). GHI is the same for natural and urban sites.

**Supplemental Tables**

**Table S1.** Genetic target qPCR assay primer and probe sequences and concentrations.

| **Assay** | **Primer/Probe sequence (5’ to 3’)^a,b^** | **Primer (Probe) Conc. (uM)** |
| --- | --- | --- |
| *Bacteroides* HF183/BacR287 | HF183: ATCATGAGTTCACATGTCCG  BacR287: CTTCCTCTCAGAACCCCTATCC  BacP234MGB: CTAATGGAACGCATCCC  Bac234IAC: AACACGCCGTTGCTACA | 1 (0.08)^c^ |
| crAssphage | f: CAGAAGTACAAACTCCTAAAAAACGTAGAG  r: GATGACCAATAAACAAGCCATTAGC  p: AATAACGATTTACGTGATGTAAC |  |
| *blaCMY* | FW3_CMY-2_Lahey: AGACGTTTAACGGCGTGTTG  RV4_CMY-2_Lahey: TAAGTGCAGCAGGCGGATAC  PR_CMY-2_Lahey: TATCGCCCGCGGCGAAAT | 0.9 (0.25) |
| *intI1* | intl1-LC1: GCCTTGATGTTACCCGAGAG  intl1-LC5: GATCGGTCG AAGCGTGT  intl1-probe: ATTCCTGGCCGTGGTTCTGGGTTTT |  |
| *KPC* | KPC_fwd: GCAGCGGCAGCAGTTTGTTGATT  KPC_rev: GTAGACGGCCAACACAATAGGTGC  KPC_probe: CAGTCGGAGACAAAACCGGAACCTGC |  |
| *MCR* | mcr1-F: CATCGCGGACAATCTCGG  mcr1-R: AAATCAACACAGGCTTTAGCAC  mcr1-P: AACAGCGTGGTGATCAGTAGCAT |  |
| *VIM* | VIM_fwd: TTGCTTTTGATTGATACAGCGTGGGG  VIM_rev: GTACGTTGCCACCCCAGCC  VIM_probe: TCTCGCGGAGATTGAAAAGCAAATTGGACTTCC |  |
| *NDM* | NDM_fwd: CCAGCAAATGGAAACTGGCGAC  NDM_rev: ATCCAGTTGAGGATCTGGGCG  NDM_probe: ACCGAATGTCTGGCAGCACACTTC |  |

^a^ Primers and probes are listed in the following order: forward (F), reverse (R), hydrolysis probe (P), and then internal amplification control probe (IAC).

^b^ Mixed bases in degenerate primers and probe are as follows: R, A or G; W, A or T. The TaqMan® probes were labeled at the 5’ end with the reporter dye FAM (6-carboxyfluorescein) or VIC™ and at the 3’ end with the quencher dye MGB (minor groove binder).

^c^ qPCR included BSA (bovine serum albumin Fraction V in phosphate-buffered saline) (Life Technologies, Grand Island, NY) at a final concentration of 0.2 mg/ml.

**Table S2**. Results of generalized additive mixed models (GAMMs) for total coliforms. *P-*values are bolded for significance (*p* < 0.05).

| **Formula: log10(Coliform) ~ s(Time, bs = "cc") + s(Time, by = Is_CS)** | | | | |
| --- | --- | --- | --- | --- |
| Family: Gaussian |  |  |  |  |
| Link function: Identity |  |  |  |  |
|  |  |  |  |  |
| Parametric coefficients: | Estimate | Std. Error | t value | Pr(>\|t\|) |
| (Intercept) | 2.336 | 0.1711 | 13.65 | **<2e-16** |
|  |  |  |  |  |
| Approximate significance of smooth terms | edf | Ref.df | F | p-value |
| s(Time) | 2.542 | 8 | 1.364 | **0.00438** |
| s(Time):Is_CS | 2 | 2 | 0.016 | 0.98462 |
|  |  |  |  |  |
| R-sq.(adj) = 0.425 |  |  |  |  |
|  |  |  |  |  |
| **Formula: log10(Coliform) ~ Water.Temp * pH * Turbidity * GHI + s(Time, bs = "cc") + s(Time, by = Is_CS)** | | | | |
| Family: Gaussian |  |  |  |  |
| Link function: Identity |  |  |  |  |
|  |  |  |  |  |
| Parametric coefficients: | Estimate | Std. Error | t value | Pr(>\|t\|) |
| (Intercept) | -1.29E+02 | NA | NA | NA |
| Water.Temp | 1.16E+01 | NA | NA | NA |
| pH | 1.63E+01 | NA | NA | NA |
| Turbidity | 2.12E+01 | NA | NA | NA |
| GHI | 4.80E-02 | NA | NA | NA |
| Water.Temp:pH | -1.44E+00 | NA | NA | NA |
| Water.Temp:Turbidity | -1.86E+00 | NA | NA | NA |
| pH:Turbidity | -2.64E+00 | NA | NA | NA |
| Water.Temp:GHI | -5.58E-03 | NA | NA | NA |
| pH:GHI | -5.21E-03 | NA | NA | NA |
| Turbidity:GHI | -1.84E-02 | NA | NA | NA |
| Water.Temp:pH:Turbidity | 2.31E-01 | NA | NA | NA |
| Water.Temp:pH:GHI | 6.32E-04 | NA | NA | NA |
| Water.Temp:Turbidity:GHI | 1.72E-03 | NA | NA | NA |
| pH:Turbidity:GHI | 2.24E-03 | NA | NA | NA |
| Water.Temp:pH:Turbidity:GHI | -2.10E-04 | NA | NA | NA |
|  |  |  |  |  |
| Approximate significance of smooth terms | edf | Ref.df | F | p-value |
| s(Time) | 1.827 | 8 | 0.863 | 0.055 |
| s(Time):Is_CS | 2 | 2 | 0.016 | 0.984 |
| R-sq.(adj) = 0.419 |  |  |  |  |

**Table S3**. Results of generalized additive mixed models (GAMMs) for *E. coli*. *P-*values are bolded for significance (*p* < 0.05).

| **Formula: Ecoli ~ s(Time, bs = "cc") + s(Time, by = Is_CS)** | | | | |
| --- | --- | --- | --- | --- |
| Family: Gaussian |  |  |  |  |
| Link function: Identity |  |  |  |  |
|  |  |  |  |  |
| Parametric coefficients | Estimate | Std. Error | t value | Pr(>\|t\|) |
| (Intercept) | 26.917 | 2.455 | 10.96 | **<2e-16** |
|  |  |  |  |  |
| Approximate significance of smooth terms | edf | Ref.df | F | p-value |
| s(Time) | 3.486 | 8 | 5.561 | **<2e-16** |
| s(Time):Is_CS | 2 | 2 | 0.917 | 0.403 |
|  |  |  |  |  |
| R-sq.(adj) = 0.669 |  |  |  |  |
|  |  |  |  |  |
| **Formula: Ecoli ~ Water.Temp * pH * Turbidity * GHI + s(Time, bs = "cc") + s(Time, by = Is_CS)** | | | | |
| Family: Gaussian |  |  |  |  |
| Link function: Identity |  |  |  |  |
|  |  |  |  |  |
| Parametric coefficients: | Estimate | Std. Error | t value | Pr(>\|t\|) |
| (Intercept) | -2.20E+03 | NA | NA | NA |
| Water.Temp | 1.85E+02 | NA | NA | NA |
| pH | 3.04E+02 | NA | NA | NA |
| Turbidity | 4.16E+02 | NA | NA | NA |
| GHI | 1.97E+00 | 2.25E+00 | 0.876 | 0.384 |
| Water.Temp:pH | -2.52E+01 | NA | NA | NA |
| Water.Temp:Turbidity | -3.33E+01 | NA | NA | NA |
| pH:Turbidity | -5.60E+01 | NA | NA | NA |
| Water.Temp:GHI | -1.81E-01 | 1.77E-01 | -1.026 | 0.308 |
| pH:GHI | -2.60E-01 | 2.85E-01 | -0.912 | 0.365 |
| Turbidity:GHI | -2.03E-01 | NA | NA | NA |
| Water.Temp:pH:Turbidity | 4.49E+00 | NA | NA | NA |
| Water.Temp:pH:GHI | 2.39E-02 | 2.24E-02 | 1.068 | 0.289 |
| Water.Temp:Turbidity:GHI | 1.71E-02 | NA | NA | NA |
| pH:Turbidity:GHI | 2.88E-02 | NA | NA | NA |
| Water.Temp:pH:Turbidity:GHI | -2.44E-03 | NA | NA | NA |
|  |  |  |  |  |
| Approximate significance of smooth terms | edf | Ref.df | F | p-value |
| s(Time) | 1.757 | 8 | 0.936 | **0.0328** |
| s(Time):Is_CS | 2 | 2 | 1.198 | 0.3076 |
| R-sq.(adj) = 0.649 |  |  |  |  |

**Table S4**. Results of generalized additive mixed models (GAMMs) for crAssphage. *P-*values are bolded for significance (*p* < 0.05).

| **Formula: log10(crAssphage) ~ s(Time, bs = "cc") + s(Time, by = Is_CS)** | | | | |
| --- | --- | --- | --- | --- |
| Family: Gaussian |  |  |  |  |
| Link function: Identity |  |  |  |  |
|  |  |  |  |  |
| Parametric coefficients: | Estimate | Std. Error | t value | Pr(>\|t\|) |
| (Intercept) | 2.4545 | 0.1291 | 19.01 | **<2e-16** |
|  |  |  |  |  |
| Approximate significance of smooth terms | edf | Ref.df | F | p-value |
| s(Time) | 1.589 | 8 | 0.424 | 0.10416 |
| s(Time):Is_CS | 2 | 2 | 5.262 | **0.00694** |
|  |  |  |  |  |
| R-sq.(adj) = 0.306 |  |  |  |  |
|  |  |  |  |  |
| **Formula: log10(crAssphage) ~ Water.Temp * pH * Turbidity * GHI + s(Time, bs = "cc") + s(Time, by = Is_CS)** | | | | |
| Family: Gaussian |  |  |  |  |
| Link function: Identity |  |  |  |  |
|  |  |  |  |  |
| Parametric coefficients: | Estimate | Std. Error | t value | Pr(>\|t\|) |
| (Intercept) | -2.34E+02 | 7.00E+01 | -3.339 | **0.001341** |
| Water.Temp | 1.81E+01 | 5.89E+00 | 3.075 | **0.002992** |
| pH | 3.26E+01 | 9.13E+00 | 3.566 | **0.000653** |
| Turbidity | 2.88E+01 | 1.20E+01 | 2.392 | **0.019405** |
| GHI | 1.90E-01 | 2.28E-01 | 0.83 | 0.409128 |
| Water.Temp:pH | -2.51E+00 | 7.62E-01 | -3.295 | **0.00154** |
| Water.Temp:Turbidity | -2.18E+00 | 1.01E+00 | -2.15 | **0.034965** |
| pH:Turbidity | -4.04E+00 | 1.57E+00 | -2.567 | **0.012359** |
| Water.Temp:GHI | -1.46E-02 | 1.87E-02 | -0.781 | 0.437425 |
| pH:GHI | -2.62E-02 | 2.97E-02 | -0.882 | 0.380777 |
| Turbidity:GHI | -6.74E-03 | 2.57E-02 | -0.262 | 0.794342 |
| Water.Temp:pH:Turbidity | 3.07E-01 | 1.31E-01 | 2.338 | **0.022207** |
| Water.Temp:pH:GHI | 2.02E-03 | 2.42E-03 | 0.837 | 0.405316 |
| Water.Temp:Turbidity:GHI | 4.05E-04 | 2.10E-03 | 0.193 | 0.847412 |
| pH:Turbidity:GHI | 1.18E-03 | 3.37E-03 | 0.351 | 0.726849 |
| Water.Temp:pH:Turbidity:GHI | -7.77E-05 | 2.74E-04 | -0.284 | 0.777097 |
|  |  |  |  |  |
| Approximate significance of smooth terms | edf | Ref.df | F | p-value |
| s(Time) | 2.473 | 8 | 2.005 | **0.000979** |
| s(Time):Is_CS | 4.455 | 4.455 | 18.823 | **<2e-16** |
| R-sq.(adj) = 0.62 |  |  |  |  |

**Table S5**. Results of generalized additive mixed models (GAMMs) for HF183/BacR287. *P-*values are bolded for significance (*p* < 0.05).

| **Formula: log10(HF183) ~ s(Time, bs = "cc") + s(Time, by = Is_CS)** | | | | |
| --- | --- | --- | --- | --- |
| Family: Gaussian |  |  |  |  |
| Link function: Identity |  |  |  |  |
|  |  |  |  |  |
| Parametric coefficients: | Estimate | Std. Error | t value | Pr(>\|t\|) |
| (Intercept) | 2.6945 | 0.1049 | 25.68 | **<2e-16** |
|  |  |  |  |  |
| Approximate significance of smooth terms | edf | Ref.df | F | p-value |
| s(Time) | 1.64E-07 | 8 | 0 | 0.865 |
| s(Time):Is_CS | 2.00E+00 | 2 | 11.1 | **4.92E-05** |
|  |  |  |  |  |
| R-sq.(adj) = 0.221 |  |  |  |  |
|  |  |  |  |  |
| **Formula: log10(HF183) ~ Water.Temp * pH * Turbidity * GHI + s(Time, bs = "cc") + s(Time, by = Is_CS)** | | | | |
| Family: Gaussian |  |  |  |  |
| Link function: Identity |  |  |  |  |
|  |  |  |  |  |
| Parametric coefficients: | Estimate | Std. Error | t value | Pr(>\|t\|) |
| (Intercept) | -3.21E+02 | 6.92E+01 | -4.634 | **1.50E-05** |
| Water.Temp | 2.48E+01 | 5.75E+00 | 4.312 | **4.92E-05** |
| pH | 4.42E+01 | 9.14E+00 | 4.837 | **7.00E-06** |
| Turbidity | 4.15E+01 | 1.26E+01 | 3.284 | **0.00156** |
| GHI | 1.93E-01 | 2.80E-01 | 0.688 | 0.49386 |
| Water.Temp:pH | -3.41E+00 | 7.53E-01 | -4.525 | **2.26E-05** |
| Water.Temp:Turbidity | -3.16E+00 | 1.05E+00 | -3.016 | **0.00351** |
| pH:Turbidity | -5.82E+00 | 1.67E+00 | -3.497 | **0.0008** |
| Water.Temp:GHI | -1.39E-02 | 2.28E-02 | -0.609 | 0.54442 |
| pH:GHI | -2.71E-02 | 3.63E-02 | -0.746 | 0.45781 |
| Turbidity:GHI | 3.67E-03 | 3.13E-02 | 0.117 | 0.90689 |
| Water.Temp:pH:Turbidity | 4.46E-01 | 1.37E-01 | 3.252 | **0.00173** |
| Water.Temp:pH:GHI | 1.98E-03 | 2.95E-03 | 0.671 | 0.50447 |
| Water.Temp:Turbidity:GHI | -5.29E-04 | 2.53E-03 | -0.209 | 0.83521 |
| pH:Turbidity:GHI | -2.23E-05 | 4.09E-03 | -0.005 | 0.99567 |
| Water.Temp:pH:Turbidity:GHI | 3.15E-05 | 3.30E-04 | 0.095 | 0.9244 |
|  |  |  |  |  |
| Approximate significance of smooth terms | edf | Ref.df | F | p-value |
| s(Time) | 5.12E-09 | 8 | 0 | 0.0746 |
| s(Time):Is_CS | 3.89E+00 | 3.885 | 20.21 | **<2e-16** |
| R-sq.(adj) = 0.45 |  |  |  |  |

**Table S6**. Results of generalized additive mixed models (GAMMs) for *blaCMY*. *P-*values are bolded for significance (*p* < 0.05).

| **Formula: log10(blaCMY) ~ s(Time, bs = "cc") + s(Time, by = Is_CS)** | | | | |
| --- | --- | --- | --- | --- |
| Family: Gaussian |  |  |  |  |
| Link function: Identity |  |  |  |  |
|  |  |  |  |  |
| Parametric coefficients: | Estimate | Std. Error | t value | Pr(>\|t\|) |
| (Intercept) | 2.1926 | 0.1937 | 11.32 | **<2e-16** |
|  |  |  |  |  |
| Approximate significance of smooth terms | edf | Ref.df | F | p-value |
| s(Time) | 1.75E+00 | 8 | 0.637 | **0.0427** |
| s(Time):Is_CS | 2.00E+00 | 2 | 4.202 | **0.0181** |
|  |  |  |  |  |
| R-sq.(adj) = 0.164 |  |  |  |  |
|  |  |  |  |  |
| **Formula: log10(blaCMY) ~ Water.Temp * pH * Turbidity * GHI + s(Time, bs = "cc") + s(Time, by = Is_CS)** | | | | |
| Family: Gaussian |  |  |  |  |
| Link function: Identity |  |  |  |  |
|  |  |  |  |  |
| Parametric coefficients: | Estimate | Std. Error | t value | Pr(>\|t\|) |
| (Intercept) | 3.72E+02 | NA | NA | NA |
| Water.Temp | -2.94E+01 | NA | NA | NA |
| pH | -4.98E+01 | NA | NA | NA |
| Turbidity | -5.81E+01 | NA | NA | NA |
| GHI | -5.75E-01 | 4.99E-01 | -1.153 | 0.25264 |
| Water.Temp:pH | 3.98E+00 | NA | NA | NA |
| Water.Temp:Turbidity | 4.64E+00 | NA | NA | NA |
| pH:Turbidity | 7.79E+00 | NA | NA | NA |
| Water.Temp:GHI | 4.55E-02 | 4.03E-02 | 1.128 | 0.26303 |
| pH:GHI | 7.77E-02 | 6.46E-02 | 1.202 | 0.23311 |
| Turbidity:GHI | 1.22E-01 | 4.40E-02 | 2.782 | **6.80E-03** |
| Water.Temp:pH:Turbidity | -6.26E-01 | NA | NA | NA |
| Water.Temp:pH:GHI | -6.16E-03 | 5.21E-03 | -1.182 | 0.24102 |
| Water.Temp:Turbidity:GHI | -9.78E-03 | 3.47E-03 | -2.818 | **6.14E-03** |
| pH:Turbidity:GHI | -1.63E-02 | 5.74E-03 | -2.833 | **5.91E-03** |
| Water.Temp:pH:Turbidity:GHI | 1.30E-03 | 4.52E-04 | 2.884 | **5.10E-03** |
|  |  |  |  |  |
| Approximate significance of smooth terms | edf | Ref.df | F | p-value |
| s(Time) | 4.28E-09 | 8 | 0 | 0.2817 |
| s(Time):Is_CS | 2.00E+00 | 2 | 4.655 | **0.0124** |
| R-sq.(adj) = 0.198 |  |  |  |  |

**Table S7**. Results of generalized additive mixed models (GAMMs) for *intI1*. *P-*values are bolded for significance (*p* < 0.05).

| **Formula: log10(intI1) ~ s(Time, bs = "cc") + s(Time, by = Is_CS)** | | | | |
| --- | --- | --- | --- | --- |
| Family: Gaussian |  |  |  |  |
| Link function: Identity |  |  |  |  |
|  |  |  |  |  |
| Parametric coefficients: | Estimate | Std. Error | t value | Pr(>\|t\|) |
| (Intercept) | 5.865 | 0.164 | 35.75 | **<2e-16** |
|  |  |  |  |  |
| Approximate significance of smooth terms | edf | Ref.df | F | p-value |
| s(Time) | 3.15E+00 | 8 | 1.691 | **0.00328** |
| s(Time):Is_CS | 2.00E+00 | 2 | 5.767 | **0.00446** |
|  |  |  |  |  |
| R-sq.(adj) = 0.238 |  |  |  |  |
|  |  |  |  |  |
| **Formula: log10(intI1) ~ Water.Temp * pH * Turbidity * GHI + s(Time, bs = "cc") + s(Time, by = Is_CS)** | | | | |
| Family: Gaussian |  |  |  |  |
| Link function: Identity |  |  |  |  |
|  |  |  |  |  |
| Parametric coefficients: | Estimate | Std. Error | t value | Pr(>\|t\|) |
| (Intercept) | -3.18E+02 | 8.96E+01 | -3.543 | **0.00069** |
| Water.Temp | 2.38E+01 | 7.40E+00 | 3.221 | **0.001905** |
| pH | 4.61E+01 | 1.20E+01 | 3.84 | **0.000258** |
| Turbidity | 5.46E+01 | 1.69E+01 | 3.226 | **0.001875** |
| GHI | -2.43E-01 | 4.62E-01 | -0.525 | 0.600936 |
| Water.Temp:pH | -3.45E+00 | 9.76E-01 | -3.531 | **0.000717** |
| Water.Temp:Turbidity | -4.12E+00 | 1.40E+00 | -2.936 | **0.004436** |
| pH:Turbidity | -7.70E+00 | 2.24E+00 | -3.438 | **0.000968** |
| Water.Temp:GHI | 2.02E-02 | 3.76E-02 | 0.538 | 0.592434 |
| pH:GHI | 3.01E-02 | 6.00E-02 | 0.502 | 0.61741 |
| Turbidity:GHI | 5.25E-02 | 4.97E-02 | 1.058 | **2.94E-01** |
| Water.Temp:pH:Turbidity | 5.86E-01 | 1.84E-01 | 3.188 | **0.002102** |
| Water.Temp:pH:GHI | -2.49E-03 | 4.87E-03 | -0.511 | 0.611112 |
| Water.Temp:Turbidity:GHI | -4.43E-03 | 4.02E-03 | -1.103 | 2.74E-01 |
| pH:Turbidity:GHI | -6.34E-03 | 6.50E-03 | -0.976 | 3.32E-01 |
| Water.Temp:pH:Turbidity:GHI | 5.35E-04 | 5.25E-04 | 1.021 | 3.11E-01 |
|  |  |  |  |  |
| Approximate significance of smooth terms | edf | Ref.df | F | p-value |
| s(Time) | 2.33E+00 | 8 | 2.042 | **0.00165** |
| s(Time):Is_CS | 2.00E+00 | 2 | 16.21 | **1.78E-06** |
| R-sq.(adj) = 0.38 |  |  |  |  |

**Table S8**. Results of generalized additive mixed models (GAMMs) for *KPC*. *P-*values are bolded for significance (*p* < 0.05).

| **Formula: log10(KPC) ~ s(Time, bs = "cc") + s(Time, by = Is_CS)** | | | | |
| --- | --- | --- | --- | --- |
| Family: Gaussian |  |  |  |  |
| Link function: Identity |  |  |  |  |
|  |  |  |  |  |
| Parametric coefficients: | Estimate | Std. Error | t value | Pr(>\|t\|) |
| (Intercept) | 2.237 | 0.1035 | 21.62 | **<2e-16** |
|  |  |  |  |  |
| Approximate significance of smooth terms | edf | Ref.df | F | p-value |
| s(Time) | 2.23E+00 | 8 | 1.387 | **0.003** |
| s(Time):Is_CS | 2.00E+00 | 2 | 2.329 | 0.103 |
|  |  |  |  |  |
| R-sq.(adj) = 0.189 |  |  |  |  |
|  |  |  |  |  |
| **Formula: log10(KPC) ~ Water.Temp * pH * Turbidity * GHI + s(Time, bs = "cc") + s(Time, by = Is_CS)** | | | | |
| Family: Gaussian |  |  |  |  |
| Link function: Identity |  |  |  |  |
|  |  |  |  |  |
| Parametric coefficients: | Estimate | Std. Error | t value | Pr(>\|t\|) |
| (Intercept) | -2.34E+02 | 6.80E+01 | -3.443 | **0.00094** |
| Water.Temp | 1.99E+01 | 5.68E+00 | 3.507 | **0.000764** |
| pH | 2.89E+01 | 9.01E+00 | 3.21 | **0.001944** |
| Turbidity | 4.21E+01 | 1.19E+01 | 3.53 | **0.000711** |
| GHI | 3.82E-01 | 3.24E-01 | 1.179 | 0.242039 |
| Water.Temp:pH | -2.43E+00 | 7.43E-01 | -3.272 | **0.001607** |
| Water.Temp:Turbidity | -3.55E+00 | 9.94E-01 | -3.571 | **0.000622** |
| pH:Turbidity | -5.23E+00 | 1.57E+00 | -3.327 | **0.001356** |
| Water.Temp:GHI | -3.06E-02 | 2.63E-02 | -1.161 | 0.249082 |
| pH:GHI | -4.73E-02 | 4.21E-02 | -1.123 | 0.265052 |
| Turbidity:GHI | -3.40E-02 | 3.52E-02 | -0.966 | 3.37E-01 |
| Water.Temp:pH:Turbidity | 4.39E-01 | 1.30E-01 | 3.384 | **0.001132** |
| Water.Temp:pH:GHI | 3.77E-03 | 3.42E-03 | 1.104 | 0.272919 |
| Water.Temp:Turbidity:GHI | 2.74E-03 | 2.85E-03 | 0.961 | 3.39E-01 |
| pH:Turbidity:GHI | 4.15E-03 | 4.61E-03 | 0.901 | 3.70E-01 |
| Water.Temp:pH:Turbidity:GHI | -3.32E-04 | 3.72E-04 | -0.89 | 3.76E-01 |
|  |  |  |  |  |
| Approximate significance of smooth terms | edf | Ref.df | F | p-value |
| s(Time) | 2.12E-09 | 8 | 0 | 0.313 |
| s(Time):Is_CS | 2.00E+00 | 2 | 2.273 | 1.10E-01 |
| R-sq.(adj) = 0.192 |  |  |  |  |

**Table S9**. Data from structured observations conducted at the recreational site between 09:00 and 14:00 on four separate weekends in April 2016.

|  | **Number of observations** | | **Percentage of total observations** |
| --- | --- | --- | --- |
| **Type of watercraft (n=36)** |  | |  |
| Kayak | 32 | | 89% |
| Boat | 4 | | 11% |
|  |  | |  |
| **Type of water interaction (n = 34)** |  | |  |
| Wading | 15 | | 42% |
| Swimming | 2 | | 6% |
| Splashing | 3 | | 8% |
| Standing In | 11 | | 31% |
| Fishing | 3 | | 8% |
|  |  | |  |
| **Head/face contact with water (n = 36)** |  | |  |
| Yes | 6 | | 17% |
| No | 30 | | 83% |
|  |  | |  |
| **Type of shoes (n = 36)** |  | |  |
| Open sandals | 11 | | 31% |
| Water shoes | 5 | | 14% |
| Close toed shoes | 13 | | 36% |
| No shoes | 3 | | 8% |
| Unknown | 4 | | 11% |
|  |  | |  |
| **Type of clothing (bottoms) (n = 36)** |  | |  |
| Pants | 16 | | 44% |
| Shorts | 16 | | 44% |
| Swimsuit | 3 | | 8% |
| Unknown | 1 | | 3% |
|  |  | |  |
| **Type of clothing (top)**  **(n = 36)** |  | |  |
| Sweater | 4 | | 11% |
| T-shirt | 14 | | 39% |
| Jacket | 13 | | 36% |
| No shirt | 1 | | 3% |
| Tank top | 2 | | 6% |
| Bathing suit | 1 | | 3% |
| Wetsuit | 1 | | 3% |
|  |  | |  |
| **Negative comments**  **(n = 36)** |  | |  |
| Yes | 9 | | 25% |
| No | 27 | | 75% |
|  |  | |  |
| **Did the person litter?**  **(n = 36)** |  | |  |
| Yes | 0 | | 0% |
| No | 36 | | 100% |
|  |  | |  |
| **Type of animal interactions (n = 4)** |  | |  |
| Swimming | 2 | | 50% |
| Standing | 1 | | 25% |
| Drinking | 1 | | 25% |
|  |  | |  |
| **Was water present on face and mouth? (n = 4)** |  | |  |
| Yes | 4 | | 100% |
| No | 0 | | 0% |
|  |  | |  |
| **Does the owner touch wet dog? (n = 4)** |  | |  |
| Yes | 3 | | 75% |
| No | 1 | | 25% |
|  |  | |  |
| **Does owner get wet? (n = 4)** |  | |  |
| Yes | 3 | | 75% |
| No | 1 | | 25% |
|  | |  |  |

1. State: GISGeography (2022) *Georgia Lakes and Rivers Map* [Map]. Available at <https://gisgeography.com/georgia-lakes-rivers-map/> (Accessed October 27, 2022).

   Sample sites: Google Maps (2022). *Chattahoochee River National Recreation Area - Paces Mill Unit.* Available at: <https://www.google.com/maps/place/Chattahoochee+River+National+Recreation+Area+-+Paces+Mill+Unit/@33.8933284,-84.4738973,13z> (Accessed October 27, 2022). [↑](#endnote-ref-1)
2. U.S. Geological Survey, 2016, National Water Information System data available on the World Wide Web (USGS Water Data for the Nation), accessed [October 17, 2022], at URL [http://waterdata.usgs.gov/nwis/]. [↑](#endnote-ref-2)
